# Quasi-Static Mechanical Properties and Continuum Constitutive Model of the Thyroid Gland

**DOI:** 10.3390/jfb13040283

**Published:** 2022-12-08

**Authors:** Peng Su, Chao Yue, Likun Cui, Qinjian Zhang, Baoguo Liu, Tian Liu

**Affiliations:** 1 College of Mechanical and Electrical Engineering, Beijing Information Science and Technology University, Beijing 110192, China; 2 Key Laboratory of Carcinogenesis and Translational Research (Ministry of Education/Beijing), Department of Head & Neck Surgery, Peking University Cancer Hospital & Institute, Beijing 100142, China

**Keywords:** thyroid, biomechanics, constitutive model, hyperelasticity

## Abstract

The purpose of this study is to obtain the digital twin parameters of the thyroid gland and to build a constitutional model of the thyroid gland based on continuum mechanics, which will lay the foundation for the establishment of a surgical training system for the thyroid surgery robot and the development of the digital twin of the thyroid gland. First, thyroid parenchyma was obtained from fresh porcine thyroid tissue and subjected to quasi-static unconfined uniaxial compression tests using a biomechanical test platform with two strain rates (0.005 s^−1^ and 0.05 s^−1^) and two loading orientations (perpendicular to the thyroid surface and parallel to the thyroid surface). Based on this, a tensile thyroid model was established to simulate the stretching process by using the finite element method. The thyroid stretching test was carried out under the same parameters to verify the validity of the hyperelastic constitutive model. The quasi-static mechanical property parameters of the thyroid tissue were obtained by a quasi-static unconstrained uniaxial compression test, and a constitutional model that can describe the quasi-static mechanical properties of thyroid tissue was proposed based on the principle of continuum media mechanics, which is of great value for the establishment of a surgical training system for the head and neck surgery robot and for the development of the thyroid digital twin.

## 1. Introduction

Thyroid cancer is a malignant tumor with a high clinical incidence, and its incidence has been increasing year by year in recent years. Surgery is a common treatment modality [1]. With the development of surgical robotics, more and more surgical robots are used in thyroid surgery; however, traditional surgical robot training has the challenges of a high cost; long lead time; high risk; difficult quantitative evaluation; and high reliance on cadavers, silicone models, and animals [2,3]. Supported by virtual reality, artificial intelligence, human–computer interaction, digital twin, modern medicine, and other technologies, establishing virtual operating area models, carrying out virtual surgery training, realizing digital simulation of the soft tissue structure and function from microscopic to macroscopic, and complete presentation of intraoperative tissue and organ morphology, rheological models are the key breakthroughs for solving the above problems.

Virtual surgical training, based on interaction and feedback, has significant advantages over traditional surgical robot training, which relies on physical objects, in terms of reducing risk costs and avoiding ethical and moral issues [4]. To establish soft tissue digital twins in virtual surgical training systems, it is urgent to understand the real mechanical properties of tissues and organs, as well as to provide accurate material constitutive models. Therefore, it is of great importance to fully understand the quasi-static mechanical properties of the thyroid gland and to develop corresponding material constitutive models.

Yamada H. [5] and Thibault L.E. et al. [6] earlier focused on the mechanical properties of soft tissue organs and studied the mechanical properties of organ soft tissues using rabbits and primates, respectively. In the last two decades, surgical robots have developed more rapidly, and their safety assessment has received more and more attention and more experimental tools have been used. Several researchers have investigated the mechanical properties of soft tissues using compression tests [7,8,9], tensile tests [10,11], and indentation tests [12,13], and there are some differences in the results obtained under different testing conditions or experimental specifications. In addition, some indirect measurement methods based on imaging techniques, such as magnetic resonance elastography (MRE) [14] and ultrasound imaging [15], have also been applied to study the mechanical properties of soft tissues. Most of these direct or indirect testing methods are for soft tissues with a larger morphology, such as liver, muscle, and tracheal hose, and mechanical properties studies on the thyroid gland have not been reported.

Biomechanical modeling of soft tissues is one of the applications of rheological models. The functional relationship between the stress tensor and strain tensor is usually referred to as constitutive model, which is a mathematical model reflecting the macroscopic properties of the material. Zahra et al. [16] proposed a hyperelastic constitutive model to describe the behavior of soft tissue (as an isotropic homogeneous material) based on continuum media mechanics. Yang et al. [17] proposed a porous hyperelastic model using the shear wave elastography (SWE) technique and, based on this constitutive model, derived the relationship between the wave velocity and the solid matrix deformation generated by the parameters of the constitutive model and the internal pressure. There are less experimental data and studies on the mechanical properties of thyroid tissues in previous studies, and there is no consultable constitutive model of the thyroid that can correspond to the experimental results.

In this paper, we investigated the quasi-static mechanical properties of porcine thyroid tissue using a biomechanical test platform equipped with Nano25 high-precision sensors, and designed quasi-static unconfined uniaxial compression tests with strain rates of 0.005 s^−1^ and 0.05 s^−1^, and loading directions perpendicular to and parallel to the thyroid surface. The effects of different strain rates and loading directions on the mechanical properties of porcine thyroid tissue were investigated. Based on this, a constitutive model was developed to describe the quasi-static mechanical properties of the porcine thyroid gland based on the theory of a hyperelastic constitutive model of a rubber-like material.

## 2. Materials and Methods

### 2.1. Specimen Preparation

Out of respect and adherence to ethical norms, animal tissues and organs are usually used as substitutes to approximate the mechanical properties of human tissues and organs, and this result will be influenced by the animal species [18], and the reference significance of choosing animals with mechanical properties closer to those of human tissues and organs for the study will be greater. Numerous scholars have tested the mechanical properties of the soft tissues of pigs and then studied the mechanical properties of human soft tissues [9,19,20]. Therefore, in this paper, the porcine thyroid gland was chosen as a substitute for studying the mechanical properties of the human thyroid gland.

The thyroid gland used in the experiment was obtained from 10 adult Landrace pigs, and the whole neck was cut and separated after slaughter and transported to the laboratory at a constant temperature of 4 °C. In order to maintain the moisture of the organ tissues, saline was sprayed regularly during the preparation of specimens and experiments after delivery to the laboratory; meanwhile, to minimize the effect of post-mortem time on tissue mechanics, each experiment was controlled within 8 h after slaughter.

**Figure 1:**
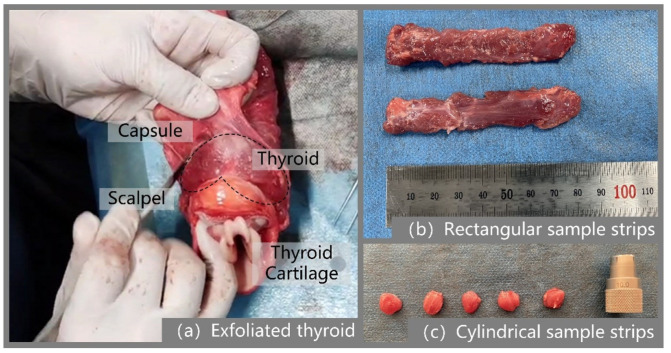
Diagram of the process for preparing thyroid specimens.

Isolation of the thyroid gland from the neck of a pig is a prerequisite for specimen preparation, a process that requires the experimenter to be able to accurately identify the thyroid gland to be isolated from the complex tissue structure of the pig neck. The location of the porcine thyroid gland is similar to that of the human body and is located at the junction of the larynx and trachea, approximately anterior to the second to fourth tracheal cartilage rings. The porcine thyroid samples in the experiment were taken from the parenchymal portion of the thyroid gland. The thyroid gland was first identified as described above, and was detached from the cervical trachea and surrounding tissues and muscles with a scalpel. A total of 10 thyroid glands were isolated, each weighing approximately 50 g. Then, a rectangular sample strip with a thickness of about 6 mm was cut out using a double-row tool, and then annular drilling tools were used to drill a circular sample strip of the thyroid gland with a diameter of about 10 mm in two directions: perpendicular to the thyroid surface and parallel to the thyroid surface, respectively, as shown in Figure 1. The prepared specimen was in the form of a cylinder with a diameter of 10 mm and a height of 6 mm. Large defects in the sample should be avoided during the preparation process, such as the inclusion of blood vessels, apparent unevenness of the upper and lower surfaces, and other parts. The original dimensions of the thyroid sample strips were measured using digital vernier calipers to measure their length, width, and thickness several times, and the average value was taken as the initial dimensions of the sample strips.

### 2.2. Uniaxial Compression Experiment

In the study of soft tissue biomechanical properties, multi-axial tensile and compression experiments are often designed. In the article, an unrestricted compression test under quasi-static conditions was designed to study the quasi-static mechanical properties of the thyroid gland. The reason for this is that considering the small size of the thyroid gland, it was difficult to prepare suitable samples for multiaxial stretching. To try to compensate for the shortcomings of the uniaxial experiments, the article prepared experimental samples from perpendicular to the surface of the porcine thyroid gland and parallel to the surface of the porcine thyroid gland, respectively, and the samples were taken from the parenchymal part of the porcine thyroid gland, avoiding the blood vessels and uneven parts of the thyroid gland as much as possible. The unconfined compression experimental method is one of the common means to obtain the mechanical response of soft tissues [18], which usually uses a compression platform to compress soft tissue samples with a certain regular shape, and the contact surface between the platform and the soft tissue sample is in a free state during the compression process, and ensures that the soft tissue sample can be deformed freely along the radial direction as much as possible.

**Figure 2:**
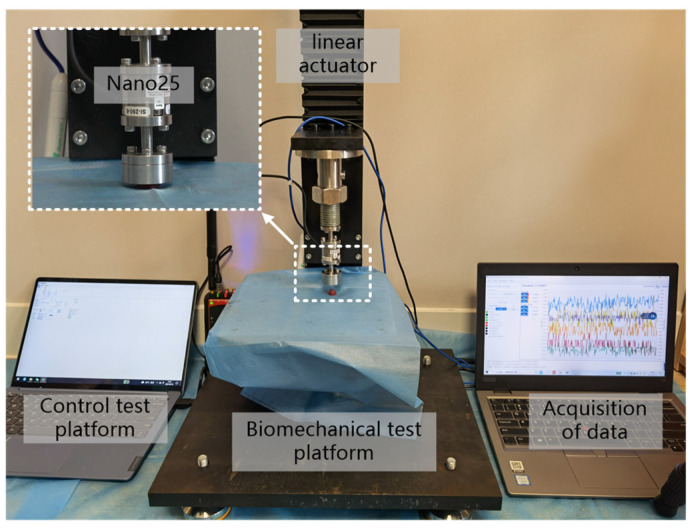
Uniaxial compression test of the thyroid gland.

The thyroid gland quasi-static compression experiments were performed on a built biomechanical test platform, as shown in Figure 2, which was equipped with a Nano25 high-precision six-dimensional force/moment transducer with a high accuracy and high stiffness, and the experimental strain rate was set at 0.005 s^−1^ and 0.05 s^−1^, in accordance with the requirements of the quasi-static tests. As the elasticity of the biological soft tissues, such as the thyroid gland, mainly comes from changes in entropy, and there is no unique natural state for it [8], in order to achieve a relatively stable state for the samples, the prepared samples were pretreated before conducting the experiments, i.e., 10 load–unload cycles of compression were performed with a compression load of 3 N each time, and the samples were left to stand for 200 s after the pretreatment as the recovery time of the samples. After the recovery phase, the thyroid sample was placed in the center of the compression lower platform, and the compression table was controlled to compress the sample at 0.03 mm/s until the strain was greater than 25%, and the parameters of pressure, displacement and time during this process were collected at 125 Hz. Ten sets of experiments were conducted for both samples (vertical thyroid surface and parallel thyroid surface) according to the above test method. During the above experiments, a small amount of saline was dropped on the sample surface at regular intervals in order to avoid excessive water loss from the sample. In addition, to meet the needs of unrestricted compression, a layer of vegetable oil (vegetable oil processed from GM soybeans with a viscosity (E020 °C) of 8.5) was brushed on the surface of the two indenters in contact with the specimen.

### 2.3. Hyperelastic Modeling

A constitutive model based on strain energy functions is commonly used to describe the hyperelastic properties of rubber-like materials, and several commonly used strain energy density functions are provided in some finite element software, such as the Mooney–Rivlin model [21,22], the Yeoh model [23,24], the Ogden model [25], and the Neo- Hookean model [26]. Based on these commonly used strain energy density functions, or with appropriate modifications, some researchers have developed constitutive models describing the hyperelastic characteristics of various types of biological soft tissues and other materials [27,28,29], and the article will also develop a constitutive model for hyperelasticity of the thyroid tissue under low strain rate conditions by choosing an appropriate strain energy density function.

According to the finite deformation theory, a material point is located at X before deformation and it is located at x after deformation, then the deformation gradient is

(1) F=∂x/∂X

In the case of finite deformation, the deformation of the material can be described by the right Cauchy–Green deformation tensor *C*. The right Cauchy–Green tensor *C* is expressed by the following equation

(2) $$C=F^{T}F$$

The effect of tissue anisotropy on the model cannot be ignored in the modeling of biological soft tissues. Giannokostas et al. [30] included the influence of the anisotropy of the parts of the arteries in the modeling of blood vessels, which greatly improved the accuracy of the model; in the modeling of organs such as the liver, kidney, and spleen [8,9,29], they are usually considered as an isotropic material to study. In the article, it is considered that the thyroid gland is a gland with internal capillaries, capsules, and other structures, similar to organs in terms of structure and somewhat different from blood vessels. In order to simplify the model as much as possible and reduce the computational effort of the model, the thyroid gland is approximated as an isotropic material.

The three invariants, $I_{1}$, $I_{2}$ and $I_{3}$, of the Cauchy–Green deformation tensor *C* for an isotropic material are denoted as

(3) $$\left\{ \begin{array}{l} I_{1}=\mathrm{tr}(C)=\lambda_{1}^{2}+\lambda_{2}^{2}+\lambda_{3}^{2}\\ I_{2}=\frac{1}{2}\left[I_{1}^{2}-\mathrm{tr}(C^{2})\right]=\lambda_{1}^{2}\lambda_{2}^{2}+\lambda_{2}^{2}\lambda_{3}^{2}+\lambda_{3}^{2}\lambda_{1}^{2}\\ I_{3}=\det(C)=\lambda_{1}^{2}\lambda_{2}^{2}\lambda_{3}^{2} \end{array}\right.$$

where $\lambda_{1}$, $\lambda_{2}$, and $\lambda_{3}$, denote the three principal elongations. When the material is incompressible, $I_{3}=1$. For incompressible isotropic materials, the strain energy density function is usually a function of the strain invariants $I_{1}$ and $I_{2}$.

The Yeoh model [23] is a cubic strain energy function, which is able to describe the materials in which the shear modulus changes with deformation, and the parameters obtained from the experimental fitting of some simple deformation can predict the mechanical behavior of other deformation cases, and the applicable deformation range is also wide enough to simulate large deformations. The model contains only the invariant variable $I_{1}$, and the strain energy density function is

(4) $$W=C_{10}\left(I_{1}-3\right)+C_{20}{\left(I_{1}-3\right)}^{2}+C_{30}{\left(I_{1}-3\right)}^{3}$$

where, $C_{10}$, $C_{20}$, and $C_{30}$ are the material parameters.

For incompressible hyperelastic materials, the principal Cauchy stress is usually determined by the following equation

(5) $$\sigma_{i}^{e}=\lambda_{i}\frac{\partial W}{\partial \lambda_{i}}-p^{e}(i=1,2,3)$$

where $p^{e}$ is the hydrostatic pressure of the hyperelastic material.

Substituting Equations (3) and (4) into Equation (5), the principal Cauchy stress can be expressed as

(6) $$\sigma_{i}^{e}=2\lambda_{i}^{2}\left[C_{10}+2C_{20}\left(I_{1}-3\right)+3C_{30}{\left(I_{1}-3\right)}^{2}\right]-p^{e}$$

For the uniaxial compression test, let $\lambda_{1}=\lambda$ denote the elongation in the loading direction and $\sigma_{1}^{e}=\sigma^{e}$ denote the first principal Cauchy stress, under the assumption of incompressibility, we have

(7) $$\left\{ \begin{array}{l} \lambda_{2}=\lambda_{3}=\lambda^{-1/2}\\ \sigma_{2}^{e}=\sigma_{3}^{e}=0 \end{array}\right.$$

Substituting the above equation into Equation (6), we have

(8) $$\sigma^{e}=2\lambda^{2}\left[C_{10}+2C_{20}\left(I_{1}-3\right)+3C_{30}{\left(I_{1}-3\right)}^{2}\right]-p^{e}$$

(9) $$0=2\lambda^{-1}\left[C_{10}+2C_{20}\left(I_{1}-3\right)+3C_{30}{\left(I_{1}-3\right)}^{2}\right]-p^{e}$$

Combining the above two equations with Equations (3) and (7) yields

(10) $$\sigma^{e}=2\left(\lambda^{2}-\lambda^{-1}\right)\left[C_{10}+2C_{20}\left(\lambda^{2}+2\lambda^{-1}-3\right)+3C_{30}{\left(\lambda^{2}+2\lambda^{-1}-3\right)}^{2}\right]$$

In the above equation, the principal elongation λ can be expressed by λ=1+ε, and ε is the engineering strain; the principal Cauchy stress $\sigma^{e}$ can be expressed by $\sigma^{e}=T^{e}\lambda$, and $T^{e}$ is the engineering stress.

The engineering stress–strain curve obtained from the quasi-static compression test with a strain rate of 0.005 s^−1^ was selected for the fitting, and parameters $C_{10}$, $C_{20}$, and $C_{30}$ were obtained.

## 3. Results and Discussion

### 3.1. Quasi-Static Mechanical Properties

In the quasi-static compression test of the porcine thyroid, the directly obtained data are the loading force, loading velocity, time, etc. The corresponding calculations using the directly obtained raw data allow for the analysis of stress–strain, stress–elongation, stress–time, strain–time, and other relationships. The relevant parameters are calculated as follows

(11) $$\sigma =\frac{F}{A}$$

(12) $$\varepsilon =\frac{L-L_{0}}{L_{0}}=\frac{\Delta L}{L_{0}}$$

where σ is the stress in MPa; *F* is the loading force applied to the soft tissue collected by the transducer in *N*; A is the initial cross-sectional area of the sample in mm^2^; ε is the strain, dimensionless; *L* is the length of the specimen after stretching in mm; and $L_{0}$ is the initial specimen length in mm.

**Figure 3:**
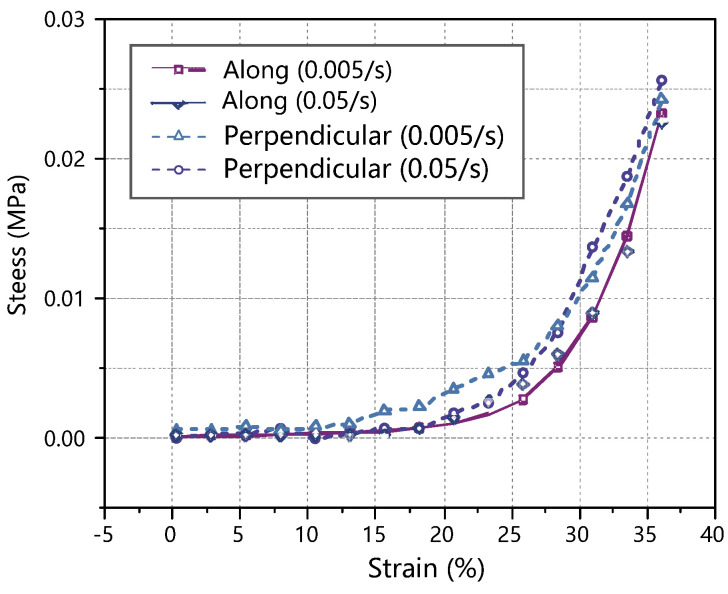
Stress–strain curve of the thyroid quasi-static compression test.

The resulting stress–strain curves were obtained for porcine thyroid tissue at two strain rates (0.005 s^−1^ and 0.05 s^−1^) and two loading directions (perpendicular to the thyroid surface and parallel to the thyroid surface). As the samples had some individual variability, the average stress at the same strain in the six sets of experiments was taken to make a stress/strain graph, as shown in Figure 3. It can be seen from the figure that the mechanical properties of the thyroid tissue did not change significantly when the strain rate increased from 0.005 s^−1^ to 0.05 s^−1^; the average stress–strain curves of the thyroid tissue were also very close for both loading directions, which indicates that the thyroid tissue can be considered as an isotropic material as far as the specimens used in the tests are concerned. Similar conclusions were reached by Farhana [8] and Umale et al. [9] in their studies on the mechanical properties of soft tissues such as the liver, kidney, and spleen.

**Figure 4:**
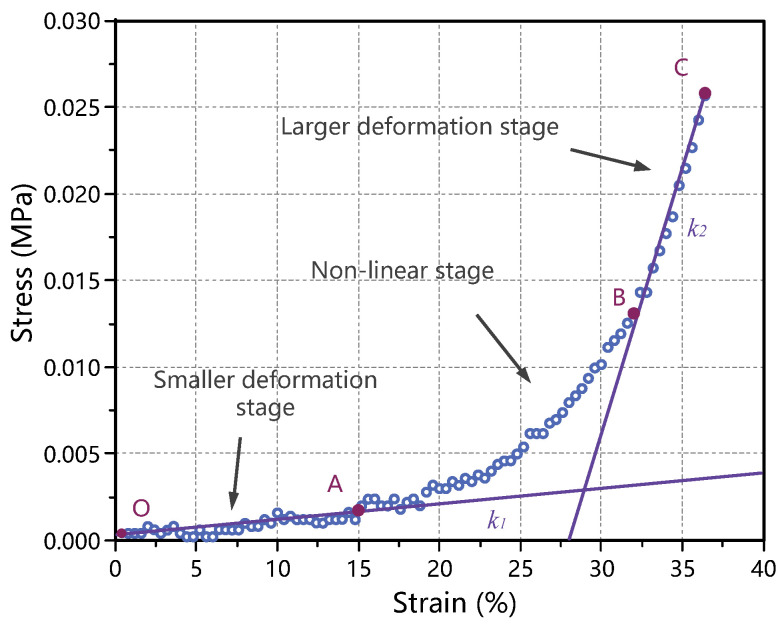
Analysis of the mechanical properties of the thyroid gland in pigs.

The average stress–strain diagram obtained from the compression test data of porcine thyroid tissue is shown in Figure 4, and the quasi-static mechanical properties of the thyroid were analyzed based on this diagram. The figure shows that the stress–strain diagram under quasi-static showed a concave-upward nonlinear characteristic, the stress amplitude was low in the initial stage, and when the strain exceeded 30%, the thyroid tissue was gradually compacted and the stress increased rapidly. The compression experimental process was divided into three stages: the first stage was the small deformation stage (segment OA in the figure), in which the stress–strain curve varied approximately linearly (slope $k_{1}$) and the stress increased slowly with the increasing strain; the second stage was the nonlinear stage (segment AB in the figure), in which the stress–strain curve showed a nonlinear variation; the third stage was the large deformation stage (segment BC in the figure), in which the stress–strain curve again showed a linear variation (slope $k_{2}$) and the stress increased rapidly with the increasing strain.

In a material stress–strain curve, the slope of the linear phase represents the Young’s modulus. For thyroid tissue, the slope of the stress–strain curve in the small deformation phase ($k_{1}$) can be used as the Young’s modulus in the small deformation phase of the thyroid ($E_{1}$), and the slope of the stress–strain curve in the large deformation phase ($k_{2}$) can be used as the Young’s modulus in the large deformation phase of the thyroid ($E_{2}$). It is of interest that in the large deformation stage, a certain degree of damage occurred inside the thyroid tissue, thus this stage contained the plastic phase of the thyroid tissue deformation process. According to the average stress–strain diagram, the Young’s modulus of the thyroid tissue in the small deformation stage and the Young’s modulus in the large deformation stage could be obtained, and the calculated results are shown in Table 1.

### 3.2. Hyperelastic Constitutive Model

**Figure 5:**
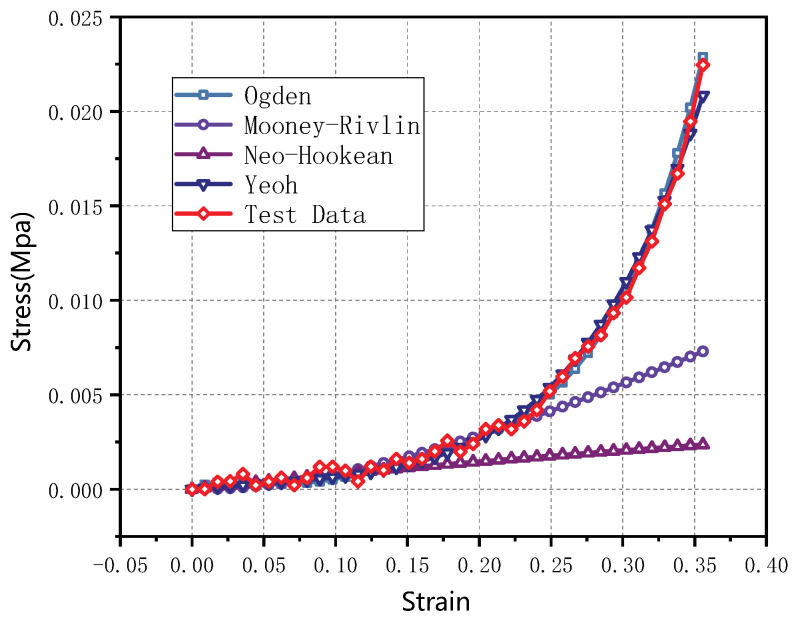
Fitting of the thyroid stress–strain curve to the constitutional model.

The hyperelastic model fitting function provided by ABAQUS finite element software was used to fit the quasi-static stress–strain curve of thyroid tissue with several common hyperelastic models (Mooney–Rivlin, Ogden, Neo-Hooke, and Yeoh), and the fitting results are shown in Figure 5 and the model parameters are shown in Table 2. The fitting results showed that the Mooney–Rivlin and Neo-Hooke models differed significantly from the experimental results when describing large strains (strains exceeding 25%), and the five-parameter Ogden and Yeoh models could better describe the quasi-static mechanical properties of the thyroid tissue. Considering the minimization of the model parameters, the Yeoh model was chosen to describe it. based on the experimental data of the compression test, the coefficients of the Yeoh model could be determined, i.e., $C_{10}=1.9\times 10^{-3}$ MPa, $C_{20}=-2.3\times 10^{-3}$ MPa and $C_{30}=0.04$ MPa. Therefore, the Yeoh hyperelastic constitutive model of the thyroid tissue could be described as follows

(13) $$W=1.9\times 10^{-3}\left(I_{1}-3\right)-2.3\times 10^{-3}{\left(I_{1}-3\right)}^{2}+0.04{\left(I_{1}-3\right)}^{3}$$

This model parameter will provide an important material basis for the simulation of thyroid gland deformation and damage assessment during the interaction of surgical instruments, which is important for the safety assessment of thyroid surgery robots.

Su et al. [31] performed viscoelastic modeling of biological soft tissues using the following viscoelastic model

(14) $$E\left(t\right)=\sum_{i=1}^{n}E_{i}e^{-t/\tau_{i}}+E_{n+1}$$

where $E_{i}$ and $\tau_{i}$ are the model parameters; n is the number of the Prony-series; $E_{n+1}$ is the static elastic modulus (i.e., the long-term relaxation modulus), and

(15) $$E_{n+1}=\frac{\sigma_{\infty }}{\varepsilon_{0}}$$

where $\sigma_{\infty }$ is residual stress and $\varepsilon_{0}$ is the constant strain.

The model describes the viscoelasticity of the material more accurately and its parameters are easier to obtain by numerical fitting based on stress relaxation tests. To obtain the parameters of this model, we designed the thyroid stress relaxation test: the sample preparation was similar to the compression experiment in the form of a cylinder with a diameter of 10 mm and a height of 6 mm. The indenter in the controlled experimental platform (shown in Figure 2) compressed the sample at a speed of 200 mm/min to 0.75 times the original length, stopped compression, and fixed the indenter position for 1000 s. Six tests were performed, and the statistics of the initial and final stresses are shown in Table 3.

**Figure 6:**
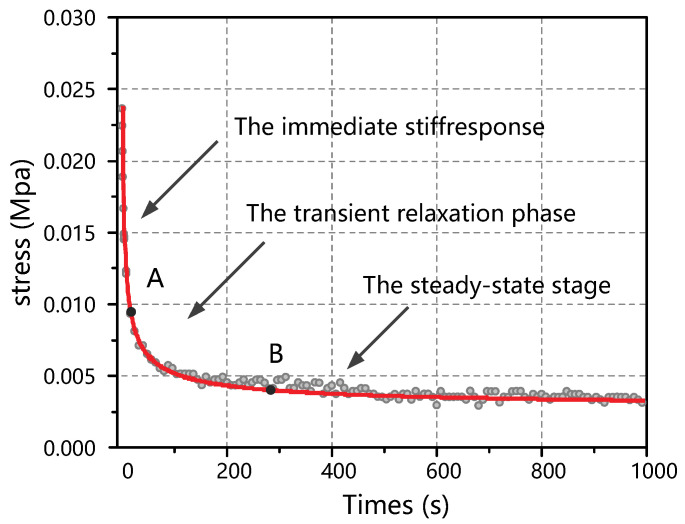
Test results corresponding to Equation (16).

It was found that the thyroid stress relaxation was accurately reflected when i=4. The mathematical expression was a relatively simple viscoelastic model. The Maxwell viscoelastic model was used to fit the thyroid stress relaxation curve, as shown in Figure 6. The model can be described as

(16) $$G\left(t\right)=\sum_{i=1}^{4}E_{i}e^{-t/\tau_{i}}+E_{5}$$

where $E_{i}$ is relaxation modulus (unit: MPa), and $\tau_{i}$ is relaxation time (unit: s), expressed by

$$\left(E_{1},E_{2},E_{3},E_{4},E_{5}\right)=\left(0.91,0.82,0.76,0.49,0.51\right)$$

And

$$\left(\tau_{1},\tau_{2},\tau_{3},\tau_{4}\right)=\left(8.83,88.68,784.29,2.89\times 10^{3}\right)$$

The results show that the behavior of the thyroid under progressive stress relaxation compression conditions consisted of an immediate stiff response, a transient relaxation phase, and a steady-state stage. In the final steady-state stage, the steady-state values reflected the residual stresses inside the thyroid gland.

### 3.3. Verification by Stretching Thyroid Specimens

**Figure 7:**
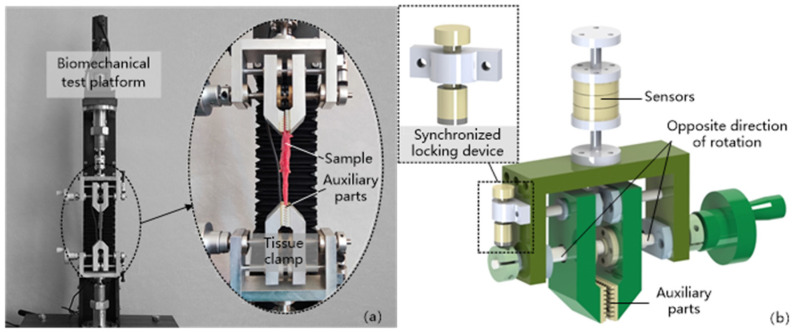
Thyroid tensile test. (**a**) Test platform and sample clamping (**b**) Tensile test jig.

Uniaxial tensile tests were performed on the porcine thyroid gland using a biomechanical test rig equipped with a Nano25 high-precision sensor, as shown in Figure 7. The test subject was the porcine thyroid gland, and the specimen was prepared as a rectangular sample strip, about 80 mm × 15 mm × 5 mm, as shown in Figure 1b, which should be left for two hours after preparation to eliminate the effect of internal stress. The use of suitable jigs in tensile experiments is one of the important factors affecting the experimental results. The current jig is not fully suitable for mechanical measurement experiments of viscoelastic materials. First, the existing jig often uses two metal clamping blocks with a certain surface roughness for clamping by threaded screwing. Secondly, there are two ways to realize the automatic centering function of the existing clamps. One is to use the restoring force of the spring and the other is to use the one-way clamping block movement. The former is not adjustable due to the non-adjustable elasticity of the spring, resulting in the clamping force of the clamps not being able to be adjusted and it is not universal for biological materials; the latter is not sufficient to maintain the force due to the one-way movement, the material can easily slip or fall off prematurely, and the stroke of the clamping block is small, which is not suitable for the clamping of large size materials. The latter is not universally applicable to biological materials because of the unidirectional motion, insufficient holding force, easy material slippage or early dislodgement, and small travel of the clamping block. Thirdly, the existing jig that can adjust the size of the clamping force lacks a synchronous locking device, and the force on the clamping block will inevitably shift during the mechanical test experiment, resulting in a change in the clamping force and the phenomenon of the material slipping or falling off prematurely. To address the above situation, a tensile test jig with automatic alignment was designed, as shown in Figure 7b. The spiral structure with the same pitch and opposite rotation direction was used to realize the synchronous anisotropic motion of the two clamping blocks, and to then realize the automatic alignment function of the jig. A device for a synchronous locking function was also added to the jig, which could lock the clamping blocks synchronously after the jig completed the clamping action. A special 3D-printed auxiliary part was designed between the jig and the specimen. The special structure of the auxiliary part could increase the friction coefficient between the biomaterial and the jig to prevent the biomaterial from slipping or falling off prematurely during the experiment. During the test, the fixture stretched the specimen at a speed of 2 mm/s until fracture, and 10 tests were conducted separately, and the relevant data such as the loading force, loading speed, and time were recorded, and the stress and strain were calculated according to Equations (11) and (12).

Then, based on the above thyroid hyperelastic model, the stretching thyroid process was simulated in an ABAQUS environment. It provides a quantitative judgment basis for the determination of the thyroid deformation. The correctness of the developed hyperelastic constitutive model was verified.

**Figure 8:**
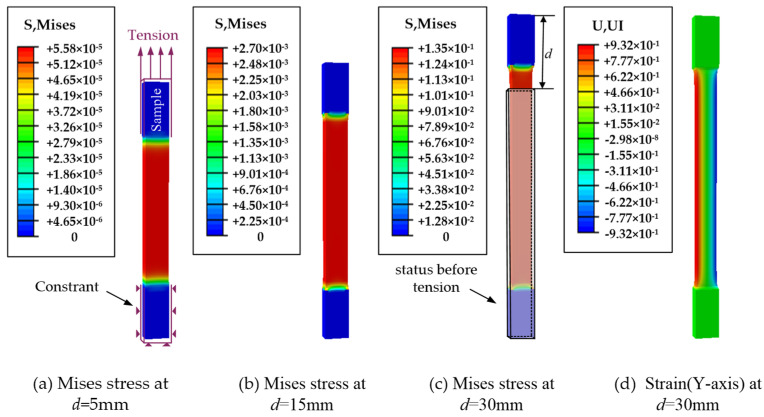
Stress–strain clouds of the tensile thyroid specimens. (**a**–**c**) show the stress clouds for tensile displacements of 5 mm, 15 mm, and 30 mm, respectively. (**d**) indicates the elastic strain cloud in the *y*-axis direction when the tensile displacement is 15 mm. In (**c**), the gray wireframe depicts the state before being stretched, where d indicates the displacement during extrusion.

- Modeling. The stretched sample model was created in ABAQUS according to the structure and dimensions (80 mm × 15 mm × 5 mm) of the thyroid sample prepared in Figure 1b.
- Properties. The thyroid material properties were defined by the hyperelastic constitutive model established above.
- Loading. The lower end of the fixed thyroid-like strip is shown in Figure 8a. The upper end of the sample was stretched at a rate of 2 mm/s.

In the simulated stretching thyroid simulation, the stress–strain cloud diagram of the sample is shown in Figure 8. At a stretching displacement of 5 mm, a small deformation of the thyroid sample occurred, as shown in Figure 8a, and the elastic strain in the *y*-axis direction was not obvious; as the stretching proceeded, the thyroid sample was gradually elongated, as shown in Figure 8b,c, and the elastic strain in the *y*-axis direction gradually increased. The elastic strain in the *y*-axis direction at a tensile displacement of 15 mm is shown in Figure 8d. Of course, there were some differences between the simulation clouds and the experimental results, because there were many factors affecting thyroid deformation, such as local deformation and uneven force. Therefore, due to the limitation of parameter setting, the simulation can only simulate the thyroid deformation under ideal conditions. The results of the uniaxial tensile test and simulated tensile test are shown in Figure 8. Obviously, in the stress–strain curves, the curves obtained from both the test and the simulation showed obvious hyperelastic characteristics. Compared with the experimental results, the slope of the stress–strain curve obtained from the simulation was larger and varied more significantly (in the large deformation phase).

**Figure 9:**
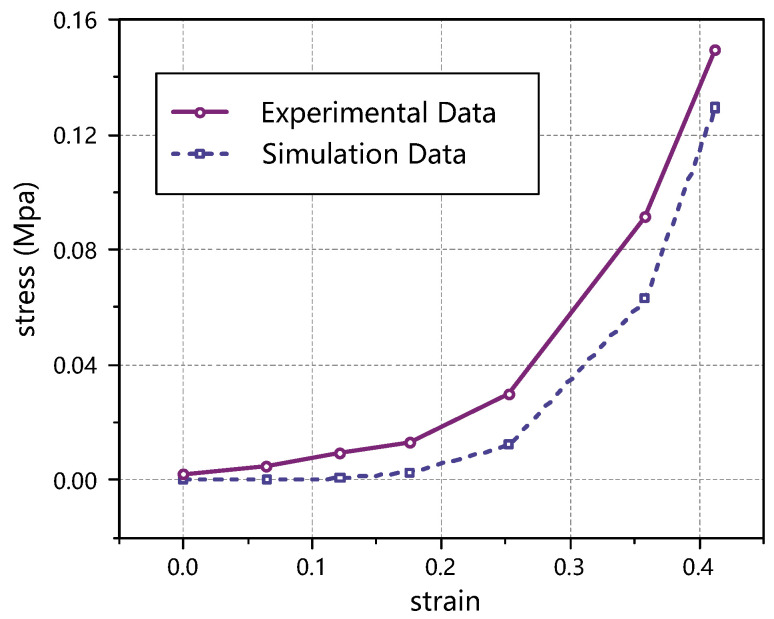
Comparison of the tensile test and simulated stress–strain.

In Figure 9, it can be seen that there is a certain difference between the simulation results and the experimental results, and in order to express the error between the simulation and experimental values, the absolute error Δ and the relative error η between them are defined as follows

(17) Δ=A−L

(18) η=Δ/L

where *A* is the simulation value and *L* is the test value. The calculated results are shown in Table 4.

The experimental data are the mean value and the simulation data are the fitting value.

The reasons for the errors are as follows. First, the sampling process of the sample strips would produce errors. As a result of the characteristics of the soft tissue, there was an error in the size of the sample in the test and the simulation model, and even if the sample was taken to avoid large blood vessels and uneven areas as much as possible, it was still difficult to avoid the existence of capillaries and other structures in the sample, which could lead to local deformation and uneven force in the test, while the simulation was performed under ideal conditions, ignoring the influence of this situation. The second is that the load setting in the simulation was not exactly the same as the actual test. In the simulation, the clamped part of the sample (the upper and lower ends of the sample) was assumed to be rigid, i.e., the clamped part was assumed not to deform in the tensile part, but the deformation of this part could not be avoided during the test. The third is the existence of extrusion in the clamping part. During the test, in order to avoid excessive water loss of the sample, saline would be applied to the sample at regular intervals, and the soft tissue itself also contained water, which would squeeze out the water in the sample during the clamping process, and the structure of the squeezed part would be changed, and this complex process is not simulated in the simulation, which will also lead to errors between the simulation results and the test results. Therefore, due to the above limitations, the simulation can only simulate the thyroid deformation under ideal conditions.

## 4. Conclusions

Uniaxial compression tests were performed on fresh porcine thyroid tissue at quasi-static strain rates of 0.005 s^−1^ and 0.05 s^−1^ and two loading directions (perpendicular to the thyroid surface and parallel to the thyroid surface) using a biomechanical test platform equipped with Nano25 high-precision sensors to investigate the effects of the strain rate and loading direction on the mechanical properties of the porcine thyroid tissue. The results showed that the porcine thyroid tissue did not exhibit significant strain rate effects in the low strain rate range of 0.005 s^−1^~0.05 s^−1^, and the loading direction had no significant effect on the mechanical properties of the porcine thyroid tissue, which can be regarded as an isotropic material. The mechanical properties of porcine thyroid were also studied under quasi-static conditions, and it was found that there was no significant difference in Young’s modulus at each stage under two strain rates and loading directions, and the average Young’s modulus was calculated to be about $2.233\times 10^{-5}$ MPa for the small deformation stage and $3.108\times 10^{-3}$ MPa for the large deformation stage of the porcine thyroid.

In order to describe the mechanical properties of porcine thyroid tissue at a quasi-static, it was considered as a non-compressible isotropic hyperelastic material, and the Yeoh strain energy density function was used to develop a hyperelastic constitutive model of porcine thyroid tissue at quasi-static low strain rate, and the three parameters of the function were obtained by fitting: $C_{10}=1.9\times 10^{-3}$ MPa, $C_{20}=-2.3\times 10^{-3}$ MPa, $C_{30}=0.04$ MPa. On the basis of this hyperelastic constitutive model, the tensile test simulation was performed on the thyroid specimens. The stress–strain curves obtained from the simulation were generally consistent with the experimental results, but some differences could be neglected because there were often some factors that could not be simulated in the actual test, such as local deformation and uneven stresses.

The quasi-static mechanical properties of the thyroid gland obtained from uniaxial unconstrained compression tests and the developed hyperelastic constitutive model are suitable for digital modeling of thyroid materials. Based on the results of this study, we are evaluating the possibility of simulating thyroid surgery and using it in a surgical training system. With the further development of virtual reality technology, we will explore methods to realize virtual thyroid surgery training by combining robotics, finite elements, and digital twins.

## Figures and Tables

**Table 1:** Results of calculating Young’s modulus of the porcine thyroid.

Test Group	$E_{1}\;(\mathrm{MPa})$	$E_{2}\;(\mathrm{MPa})$
1	$2.238\times 10^{-5}$	$3.430\times 10^{-3}$
2	$1.696\times 10^{-5}$	$3.590\times 10^{-3}$
3	$2.459\times 10^{-5}$	$2.680\times 10^{-3}$
4	$2.670\times 10^{-5}$	$2.950\times 10^{-3}$
5	$2.362\times 10^{-5}$	$2.740\times 10^{-3}$
6	$1.972\times 10^{-5}$	$3.260\times 10^{-3}$
Mean	$2.233\times 10^{-5}$	$3.108\times 10^{-3}$
Variance	$1.230\times 10^{-11}$	$1.405\times 10^{-7}$

$E_{1}$ Young’s modulus in the small deformation phase of the thyroid; $E_{2}$ Young’s modulus in the large deformation phase of the thyroid.

**Table 2:** Hyperelasticity constitutive model fitting parameters.

	$C_{10}/\mathrm{MPa}$	$C_{20}/\mathrm{MPa}$	$C_{30}/\mathrm{MPa}$	$A_{1}/\mathrm{MPa}$	$A_{2}/\mathrm{MPa}$	$R^{2}$
N-H	2.357	-	-	-	-	0.564
M-R	$1.7\times 10^{-2}$	$1.7\times 10^{-2}$	-	-	-	0.661
Ogden	−0.305	0.297	1.619	10.267	−25	0.996
Yeoh	$1.9\times 10^{-3}$	$-2.3\times 10^{-3}$	0.04	-	-	0.999

In the table, $C_{10}$, $C_{20}$, $C_{30}$, $A_{1}$ and $A_{2}$ are the material parameters; $R^{2}$ is the correlation coefficient; and “-” represents no parameter value.

**Table 3:** Statistics on stress in stress-relaxation tests.

Test Group	Initial Stress (MPa)	Last Stress (MPa)
1	$2.646\times 10^{-2}$	$3.738\times 10^{-3}$
2	$2.854\times 10^{-2}$	$4.012\times 10^{-3}$
3	$1.762\times 10^{-2}$	$3.532\times 10^{-3}$
4	$2.931\times 10^{-2}$	$4.216\times 10^{-3}$
5	$1.912\times 10^{-2}$	$2.974\times 10^{-3}$
6	$2.491\times 10^{-2}$	$3.685\times 10^{-3}$
Mean	$2.433\times 10^{-2}$	$3.693\times 10^{-3}$
Variance	$2.39\times 10^{-5}$	$1.84\times 10^{-7}$

**Table 4:** Error between the simulation and test values.

Strain	Stress (MPa)		Absolute Error (MPa)	Relative Error (%)
	Simulation Data	Experimental Data		
0	0	0	0	0
0.064	$5.576\times 10^{-5}$	$6.650\times 10^{-5}$	$-1.074\times 10^{-5}$	16.16
0.121	$6.477\times 10^{-4}$	$8.300\times 10^{-4}$	$-1.823\times 10^{-4}$	21.97
0.175	$2.690\times 10^{-3}$	$4.301\times 10^{-3}$	$-1.611\times 10^{-3}$	37.46
0.253	$1.227\times 10^{-2}$	$2.077\times 10^{-2}$	−0.0085	40.92
0.388	$6.298\times 10^{-2}$	$7.414\times 10^{-2}$	−0.0112	15.05
0.413	0.1294	0.1489	−0.0196	13.13
Mean error				20.67

## Data Availability

Not applicable.
